# Research and Remedies

**Published:** 1915-05-08

**Authors:** 


### RESEARCH AND REMEDIES.

### Isolation Hospitals and Cerebro-spinal Meningitis.

The presence of cerebro-spinal meningitis in the country at the present time has increased the responsibilities of the authorities of isolation hospitals, and has augmented the work of their staffs. In some districts, owing to various reasons, difficulties have arisen in securing the admission of these cases to isolation hospitals. Evidence is not lacking that extreme fear of this disease is not altogether restricted to the public, and it is therefore desirable to emphasise the fact that the experience of the last few months tends to the conclusion that cerebro-spinal miningitis is a disease of relatively low infectivity. Amongst the civil population, at least, one seldom if ever meets with a case which has been in direct contact with a previous case of the disease. Moreover, while the post-nasal swabs of contacts may show the presence of Gram-negative diplococci in a relatively high percentage of cases, it is only a small percentage of these organisms which will pass the specific tests for the meningococcus. The meningococcus readily succumbs to the influence of fresh air and sunlight, and cases of cerebro-spinal meningitis should therefore be treated under open air conditions wherever possible. Any risk to the members of the staff can be minimised by a few simple precautions. We know little of the life-history of the meningococcus or of the manner in which the disease spreads, but it does not appear to be air-borne. The infection is probably conveyed by hand or handkerchief to mouth or nose; hence the importance of disinfecting the hands and of not touching the face when in the sick-room. Exercise in the open air and the use of boric powder as a snuff and of 1-1000 permanganate of potassium solution as a gargle, complete the necessary precautions. The treatment of cerebro-spinal meningitis demands lumbar puncture and the intra-spinal injections of anti-meningococcic serum. Recent evidence pointing to the presence of the organism in the blood stream suggests, in addition, the advisability of subcutaneous injections.

### Puerperal Septicæmia.

Apart from local and operative measures a good influence in this condition seems to be exercised by magnesium sulphate injected intravenously. As much as 250 grains in a 1 per cent. solution has been given, but 100 grains may be considered as sufficient for a first dose; the administration may be repeated at intervals of twenty-four hours. Distilled water should be used as the solvent, and of course the fluid should be at an appropriate temperature.—*New York State Journal of Medicine*, December, 1914.

### Sphygmometer Records in Relation to Life Insurance.

A contribution by Dr. J. W. Fisher on this subject is of general interest as bearing on the clinical significance and prognostic value of the maximum sphygmometer record, or, as it is often named, systolic blood pressure. The value of the paper is all the greater seeing that it is based on a very extensive survey, which included nearly 20,000 examples of accepted lives of ages ranging from fifteen to sixty years. First, as to the standard on which the normal level is to be fixed, the figures give a decidedly lower level than that generally quoted. Leaving fractions out of account, the figure in ages fifteen to twenty is just under 120 mm. of mercury; in ages twenty-six to thirty it is 123; at thirty-six to forty years it is 127; at forty-six to fifty it is 130; and even at fifty-six to sixty (1,100 cases) the average has only risen to 134. Dr. Fisher concludes (1) that a persistent systolic pressure of 15 mm. above the average for any given age should excite suspicion and calls for careful inquiry and investigation; (2) that applicants of all ages with a persistent systolic pressure above 150 mm. show a mortality higher than normal, the rate of mortality increasing in proportion to the excess of pressure. These figures are certainly very impressive, and are the more to be noted seeing that the present tendency is to belittle the value of sphygmometer readings. At first these readings were perhaps treated with undue respect, but the pendulum of opinion has now swung to the other extreme.

In a paper dealing with the treatment of high sphygmometer readings, Dr. Robert H. Babcock insists on the value of dietetic and general measures, and on the minor rôle played by medicines. The iodides have long enjoyed considerable reputation in such cases, and small doses continued over long periods seem to be beneficial, but they do not affect the sphygmometer records. No doubt the nitrites—for example, sodium nitrite—reduce the level for a time, but their action is very short-lived, one to two hours at the outside; and there is reason to believe that even this brief influence is not continued as the patient becomes accustomed to the medicine.—*Louisville Journal of Medicine and Surgery*, January and February, 1915.

